# The diagnostic role of T wave morphology biomarkers in congenital and acquired long QT syndrome: A systematic review

**DOI:** 10.1111/anec.13015

**Published:** 2022-11-07

**Authors:** Daniel T. Tardo, Matthew Peck, Rajesh N. Subbiah, Jamie I. Vandenberg, Adam. P. Hill

**Affiliations:** ^1^ Cardiac Electrophysiology Laboratory Victor Chang Cardiac Research Institute Darlinghurst New South Wales Australia; ^2^ Department of Cardiology St. Vincent's Hospital Darlinghurst New South Wales Australia; ^3^ School of Medicine University of Notre Dame Australia Darlinghurst New South Wales Australia; ^4^ St. Vincent's Clinical School, Faculty of Medicine University of New South Wales Sydney New South Wales Australia

**Keywords:** acquired long QT syndrome, congenital long QT syndrome, ECG biomarkers, LQTS, sudden cardiac death, T wave morphology

## Abstract

**Introduction:**

QTc prolongation is key in diagnosing long QT syndrome (LQTS), however 25%–50% with congenital LQTS (cLQTS) demonstrate a normal resting QTc. T wave morphology (TWM) can distinguish cLQTS subtypes but its role in acquired LQTS (aLQTS) is unclear.

**Methods:**

Electronic databases were searched using the terms “LQTS,” “long QT syndrome,” “QTc prolongation,” “prolonged QT,” and “T wave,” “T wave morphology,” “T wave pattern,” “T wave biomarkers.” Whole text articles assessing TWM, independent of QTc, were included.

**Results:**

Seventeen studies met criteria. TWM measurements included T‐wave amplitude, duration, magnitude, Tpeak‐Tend, QTpeak, left and right slope, center of gravity (COG), sigmoidal and polynomial classifiers, repolarizing integral, morphology combination score (MCS) and principal component analysis (PCA); and vectorcardiographic biomarkers. cLQTS were distinguished from controls by sigmoidal and polynomial classifiers, MCS, QTpeak, Tpeak‐Tend, left slope; and COG *x* axis. MCS detected aLQTS more significantly than QTc. Flatness, asymmetry and notching, J‐Tpeak; and Tpeak‐Tend correlated with QTc in aLQTS. Multichannel block in aLQTS was identified by early repolarization (ERD_30%_) and late repolarization (LRD_30%_), with ERD reflecting hERG‐specific blockade. Cardiac events were predicted in cLQTS by T wave flatness, notching, and inversion in leads II and V_5_, left slope in lead V_6_; and COG last 25% in lead I. T wave right slope in lead I and T‐roundness achieved this in aLQTS.

**Conclusion:**

Numerous TWM biomarkers which supplement QTc assessment were identified. Their diagnostic capabilities include differentiation of genotypes, identification of concealed LQTS, differentiating aLQTS from cLQTS; and determining multichannel versus hERG channel blockade.

## Graphical abstract

Numerous T wave morphology biomarkers, which can supplement QTc assessment in long QT syndrome (LQTS), have been identified. Their diagnostic capabilities include differentiation of genotypes, identification of concealed LQTS, differentiating acquired LQTS from congenital LQTS; and determining multichannel versus hERG channel blockade.

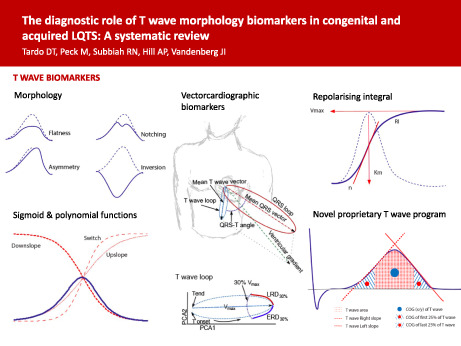

## 1 INTRODUCTION

Long QT syndrome (LQTS) is a cardiac disorder of myocardial repolarization existing as two primary syndromes, congenital (cLQTS; i.e., familial) or acquired (aLQTS). The familial form is diagnosed based on symptoms, electrocardiogram (ECG), and family history. Diagnostic criteria have been codified by Peter Schwartz, combining clinical features with family history and abnormalities of repolarization on the ECG, to provide a low (≤1 point), intermediate (1.5 to 3 points), or high (≥3.5 points) probability of LQTS (Schwartz & Crotti, 2011). Symptoms include syncope, resuscitated sudden cardiac arrest, or sudden cardiac death (SCD), often associated with exercise, emotional stress, loud noise; or sleep events (Schwartz et al., 2001). Specific ECG changes forming part of the diagnostic criteria include QTc (corrected QT interval) prolongation (categorized by ≥480 milliseconds [ms], 460–479 ms for females; and 450–459 for males), QTc ≥480 ms 4 minutes post exercise stress test, Torsades de pointes (TdP), T wave (TW) alternans, notched TW in three leads; and low heart rate for age (below the second percentile; Schwartz & Crotti, 2011).

Prior to discovering the genetic causes of cLQTS, approximately 50% of families with cLQTS were identified subsequent to the death of the proband case (Schwartz et al., 2001). With increasing awareness of cLQTS, contemporary measures indicate 27% of individuals are symptomatic at the time of presentation with a median age of onset of 12 years (Rohatgi et al., 2017). Although most probands are symptomatic when diagnosed, asymptomatic individuals are diagnosed more frequently in populations undergoing increased screening, including affected relatives of the index case. One of the big challenges surrounding the diagnosis of cLQTS is that between 25% and 50% of genotype positive individuals present with concealed LQTS, that is they have a normal resting QTc (Goldenberg et al., 2011; Immanuel et al., 2016; Sugrue et al., 2016; Sy et al., 2011). Provocation tests which can be utilized to facilitate diagnosing LQTS include an exercise test (Sy et al., 2011), as per the Schwartz score ECG criteria outlined above, and standing ECG which can enhance QTc prolongation, TW alternans or genotype‐specific TW changes (Viskin et al., 2010; Waddell‐Smith et al., 2017; Waddell‐Smith & Skinner, 2016).

There are multiple subtypes of cLQTS with the three most common subtypes being LQT1, caused by mutations in *KCNQ1* (accounts for ~45% of genotyped cases); LQT2, caused by mutations in *KCNH2* (otherwise known as the human ether‐a‐go‐go related gene, *hERG*), which accounts for ~40% of genotyped cases and LQT3, caused by mutations in *SCN5A*, which accounts for ~10% of genotyped cases. These three common subtypes can be differentiated by characteristic TW morphology (TWM) patterns. These include early onset broad TWs in LQT1 (*KCNQ1*), bifid (notched) TWs in LQT2 (*KCNH2*), and late onset TWs in LQT3 (*SCN5A*; Moss et al., 1995). These TW patterns can be subtle and go unnoticed, hence there has been considerable interest in developing methods for accurate quantitative analysis of the TW to detect repolarization abnormalities (Immanuel et al., 2016; Porta‐Sanchez et al., 2017; Sugrue et al., 2016).

The acquired form of LQTS is diagnosed on the basis of a QTc which exceeds 500 ms or when the QTc increases by >60 to 70 ms in the presence of a medication, known as drug‐induced LQTS (diLQTS), or another associated clinical precipitant (Giudicessi et al., 2020; Indraratna et al., 2019; Roden, 2004; Schwartz & Woosley, 2016). Such QTc prolonging factors include, but are not limited to, electrolyte derangements, pheochromocytoma, autonomic failure, stroke, bradycardia, takotsubo cardiomyopathy, cardiac disease, hypothyroidism, hyperparathyroidism, hypothermia, female sex, age > 65 years, grapefruit juice; and an underlying inflammatory disorder or state (Credible Meds, 2020; Roden, 2004; Schwartz & Woosley, 2016; Woosley et al., 2020). Acquired factors have a summative effect with other environmental factors and genotype factors (Al‐Khatib et al., 2018; Schwartz & Woosley, 2016; Weeke et al., 2019). Personalized risk stratification for diLQTS is aided by the Pro‐QTc score and Tisdale score diagnostic tools (Haugaa et al., 2013; Schwartz & Woosley, 2016; Tisdale et al., 2013). TWM has also been shown to enhance discrimination of abnormal repolarization in aLQTS (Couderc et al., 2011; Graff et al., 2009; Graff et al., 2010; Heijman & Crijns, 2015; Johannessen et al., 2014; Sugrue et al., 2015; Sugrue, Noseworthy, et al., 2017; Vicente et al., 2015).

In light of emerging evidence for the role of various TWM biomarkers in the diagnosis and risk stratification of LQTS, the purpose of this systematic review was to assess the knowledge in this area, including specific methodologies of TWM analysis, TW biomarkers used to identify LQTS subtypes and assess torsadogenic risk; and clarify directions for future research.

## 2 METHODS

### 2.1 Search strategy

Electronic database searches were performed using CINAHL, MEDLINE, PubMed, ScienceDirect, SPORTDiscus, and Web of Science, from the dates of their inception to 8th of July 2018 and were updated in March 2020. The search terms “LQTS,” “long QT syndrome,” “QTc prolongation,” and “prolonged QT” were combined using “OR,” and the terms “T wave,” “T wave morphology,” “T wave pattern,” “T wave biomarkers” too were combined with “OR.” Results from these two searches were combined using “AND.” Manual searching of retrieved article reference lists and related citation indexes were performed, as well as author searches of retrieved articles. Two reviewers (D.T. and M.P.) independently located records and extracted data. Full‐text publications were reviewed independently if either reviewer considered the manuscript eligible for inclusion. Resolution of disputes was mediated by means of consensus with a third reviewer (A.H.).

### 2.2 Eligibility criteria

Studies were included if they reported on the assessment of TWM in cLQTS or aLQTS, independent of QTc. All publications were full‐length, peer‐reviewed articles limited to human subjects, and written in English. Studies were not restricted by study design, subject characteristics, or methodology surrounding TWM analysis. Studies assessing single components of the TW only or describing simple architectural TW changes secondary to an intervention were excluded. The following publications were excluded: case reports, conference abstracts, editorials, expert opinions and commentaries; and textbook chapters.

## 3 RESULTS

### 3.1 Studies retrieved

**FIGURE 1:**
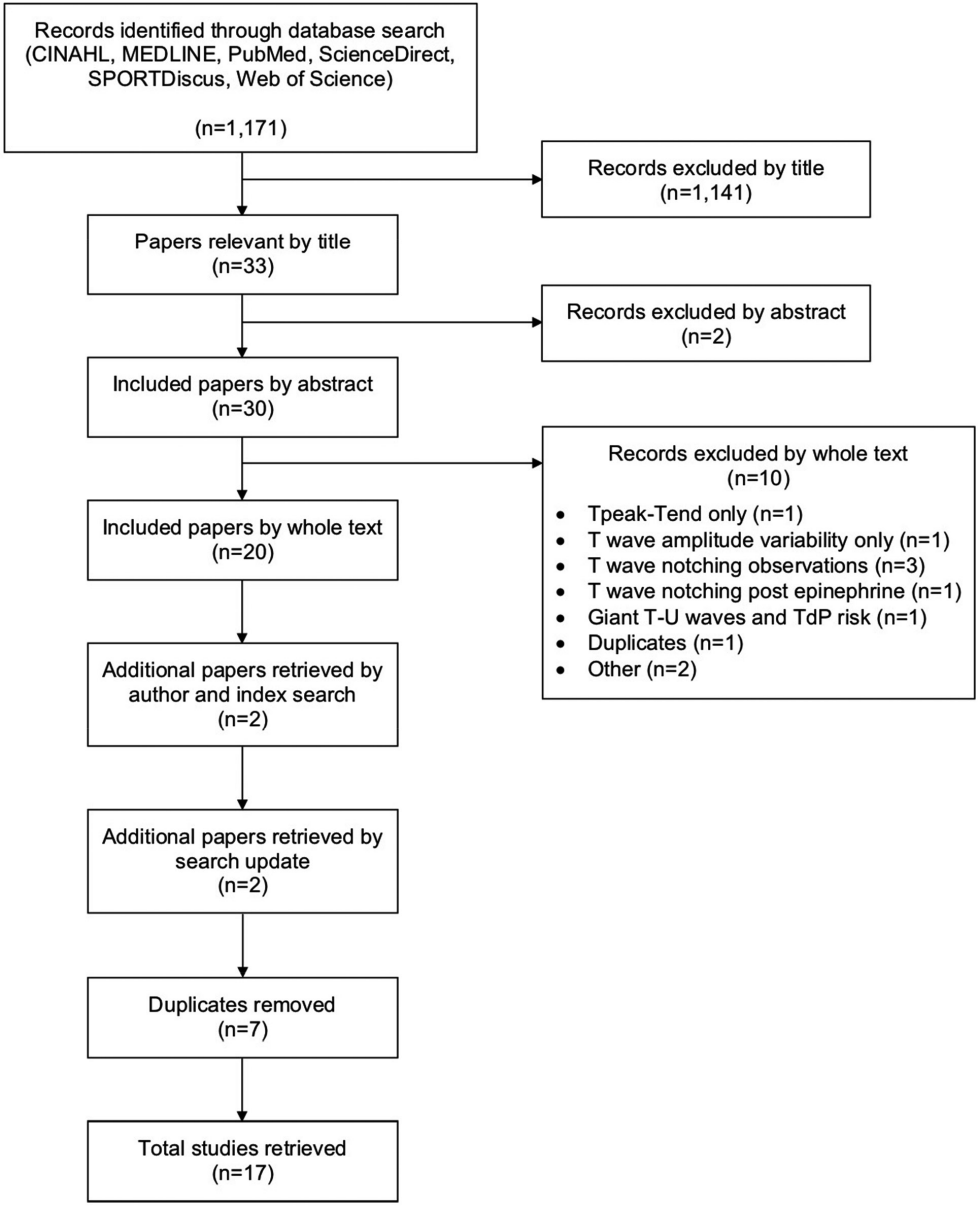
Summary of search results. TdP, Torsades de pointes

Search results are summarized in Figure 1 as per the PRISMA statement (Moher et al., 2009). The literature search identified 1171 studies across six databases. Examination of titles excluded 1141 studies, with 30 abstracts reviewed manually and a further 10 studies were excluded. Removal of seven duplicates and addition of two studies retrieved by author and citation index search, and a further two studies by search update, identified the whole text of 17 studies which met the criteria for review (Tables 1, 2, 3).

**TABLE 1:** Subject characteristics, inclusion, and exclusion criteria.

Citation	Sample size	Age (years)	Sex	Health status, LQTS type & QTc (ms)	Inclusion & exclusion criteria
Hermans et al. (2020)	Study cohort (Amsterdam): n = 678 Control = 345 cLQTS = 333 External cohort (Leuven): *n* = 117 Control = 45 cLQTS = 72	Amsterdam: 45 ± 15 (control) 42 ± 15 (LQT1) 42 ± 15 (LQT2) 40 ± 15 (LQT3) Leuven: 42.8 ± 16.6 (control) 44.3 ± 9.4 (LQT1) 35.7 ± 15 (LQT2) 34.8 ± 10.2 (LQT3)	Amsterdam: F: 185 (control) 77 (LQT1) 87 (LQT2) 32 (LQT3) M: 160 (control) 49 (LQT1) 69 (LQT2) 19 (LQT3) Leuven: F: 27 (control) 12 (LQT1) 23 (LQT2) 5 (LQT3) M: 18 (control) 4 (LQT1) 28 (LQT2) 0 (LQT3)	Amsterdam: Control (410 ± 28) LQT1 (455 ± 34) LQT2 (462 ± 36) LQT3 (446 ± 50) Leuven: Control (402 ± 27) LQT1 (467 ± 44) LQT2 (455 ± 34) LQT3 (421 ± 11)	Age ≥ 16 years Known genetic testing results Digitally available ECG at first presentation **Exclusion:** age < 16 years, absence genetic testing results, absence baseline data, pathologies and medications that affect TWM
Hermans et al. (2018)	*n* = 688 Control = 348 LQT1 = 129 LQT2 = 160 LQT3 = 51	45 ± 15 (control) 42 ± 15 (LQT1) 42 ± 15 (LQT2) 40 ± 15 (LQT3)	F: 185 (control) 77 (LQT1) 88 (LQT2) 32 (LQT3) M: 163 (control) 52 (LQT1) 72 (LQT2) 19 (LQT3)	Control LQT1 LQT2 LQT3 QTc‐interval cut‐off: >480	Age ≥ 16 years Known genetic testing results Digitally available ECG at first presentation **Exclusion:** comorbidity affecting ventricular re‐ and/or depolarization (BBB hypokalemia, thalassemia, angina pectoris, BrS overlap, severe post‐anoxic encephalopathy), ECG parameters (excessive noise, TW flattening <40 μV, export failure)
Platonov et al. (2018)	*n* = 1161 Control = 1007 LQT2 = 154	41 ± 15	F: 593 (control) 87 (LQT2) M: 414 (control) 67 (LQT2)	Control (417 ± 26) LQT2 with normal QTc (436 ± 23)	Rochester‐LQTS registry KCNH2 mutation (LQT2) QTc <470 ms (F), <460 ms (M) ≤18 years **Exclusion:** ≥1 mutation
Porta‐Sanchez et al. (2017)	*n* = 108 Control = 45 LQT1 = 43 LQT2 = 20	35.4 ± 17.3 (control) 41.7 ± 17.4 (LQTS)	F: 66.7% (control) 67.4% (LQT1) 60% (LQT2) M: 33.3% (control) 32.6% (LQT1) 40% (LQT2)	Control (418 ± 24) LQT1 (486 ± 50) LQT2 (479 ± 36)	No QT prolonging drugs No reversible causes QTc prolongation LQTS: gene positive Control: normal ECG, echocardiogram, cardiology review
Sugrue, Noseworthy, et al. (2017)	*n* = 152 LQT1 = 15 LQT2 = 23 aLQTS = 114	15 ± 12 (cLQTS) 66 ± 14 (aLQTS)	F: 30 (75%, cLQTS) 69 (53%, aLQTS) M: 8 (25%, cLQTS) 45 (47%, aLQTS)	cLQTS (500 ± 30) aLQTS (520 ± 29)	Mayo Clinic's QT‐alert system cLQTS *CredibleMeds* QT drug list (≤7 days) Hypokalemia <3.6 mm/L Hypomagnesemia <1.7 mg/dL Hypocalcemia <4.65 mg/dL (ionized) **Exclusion:** BBB, ventricular pacing, AF, atrial flutter, SVT, ST‐T ischemic changes, LVH, uninterpretable ECG, tracing interference, biphasic TW
Sugrue, Rohatgl, et al. (2017)	*n* = 491 LQT1 = 246 LQT2 = 161	16 (median age at first Mayo clinic ECG)	F: 235 (85%) M: 172 (42%)	LQT1 (456.5) LQT2 (455)	Mayo Clinic LQT cohort (1999–2015) Genotype positive LQT1, LQT2 **Exclusion:** LQT3, LQT4, multiple LQTS‐associated mutations, BBB, ventricular pacing, AF, uninterpretable ECG, biphasic TW, missing ECG lead data
Immanuel et al. (2016)	*n* = 419 Control = 159 LQT1 = 171 LQT2 = 89	35.6 ± 14.6 (control) 28.2 ± 17.7 (LQT1) 28.6 ± 18.7 (LQT2)	F: 75 (control) 78 (LQT1) 29 (LQT2) M: 65 (control) 55 (LQT1) 32 (LQT2)	Control LQT1 LQT2 Subgroup with normal QTc (400–450)	THEW database Children and adults Genotype positive LQT1, LQT2 Upright TWs **Exclusion:** abnormal TWs (flat, biphasic)
Sugrue et al. (2016)	*n* = 840 Control = 420 LQT1 = 257 LQT2 = 163	22 ± 16 (control) 23 ± 16 (LQT1) 22 ± 15 (LQT2)	F: (57%) M: (43%)	Control (424 ± 18) LQT1 (462 ± 37) LQT2 (464 ± 46)	Control: no cardiac disease Genotype positive LQT1, LQT2 Concealed LQTS: QTc <460 ms (F), <450 ms (M), <440 ms (children, both sexes) **Exclusion:** uninterpretable ECG, tracing interference, biphasic or low amplitude TW
Sugrue et al. (2015)	*n* = 39 Control = 26 TdP = 13 Sotalol = 8 Dofetilide = 5	60.3 ± 14.5 (sotalol) Control = 61.4 ± 14 68.4 ± 5.5 (dofetilide) Control = 68 ± 5.4	F: 20 (51%, control) Sotalol control = 12 Dofetilide control = 8 6 (15%, sotalol) 4 (10%, dofetilide) M: 6 (15%, control) Sotalol control = 4 Dofetilide control = 2 2 (5%, sotalol) 1 (2.6%, dofetilide)	Control TdP post drug initiation	Electronic medical record search Admitted for initiation of sotalol or dofetilide (AF, atrial flutter, VE, VT) Serial ECGs Documentation of TdP No previous TdP **Exclusion:** paced rhythm, drug ceased due to QT prolongation, chronic use of drug
Vicente et al. (2015)	*n* = 22	26.9 ± 5.5	F: 11 (50%) M: 11 (50%)	Healthy (395.9 ± 17.1)	Healthy: physician assessment, no history of heart disease or unexplained syncope or a family history of LQTS QTc (Fridericia) <450 ms (M), <470 ms (F) 18–35 years of age Weight ≥ 50 kg BMI 18–27 kg/ m^2^ Able to read and understand the informed consent **Exclusion:** >10 ectopic beats (3 hr continuous ECG recording at screening)
Johannessen et al. (2014)	*n* = 22	26.9 ± 5.5	F: 11 (50%) M: 11 (50%)	Healthy (395.9 ± 17.1)	Healthy: physician assessment, no history of heart disease or unexplained syncope or a family history of LQTS QTc <450 ms (M), <470 ms (F) 18–35 years of age Weight ≥ 50 kg BMI 18–27 kg/ m^2^ Able to read and understand the informed consent **Exclusion:** >10 ectopic beats at screening (3 hr continuous ECG)
Couderc et al. (2011)	*n* = 704 Control = 411 LQT2 Noncarrier = 150 Carrier = 143	40 ± 14 (control) 39 ± 14 (LQT2 noncarrier) 38 ± 15 (LQT2 carrier)	F: 267 (65%, control) 86 (57%, LQT2 noncarrier) 87 (61%, LQT2 carrier) M: 144 (35%, control) 64 (43%, LQT2 noncarrier) 56 (39%, LQT2 carrier)	Control (411 ± 23) Healthy on moxifloxacin (422 ± 26) LQT2 Noncarrier (405 ± 29) Carrier (470 ± 47)	Age > 17 years LQT2 families Adequate quality ECG trace
Graff et al. (2010)	*n* = 145 Placebo = 62 Moxifloxacin = 62 Sotalol = 21	18 to 45	F: 26 (placebo) 24 (moxifloxacin) 0 (sotalol) M: 36 (placebo) 38 (moxifloxacin) 21 (sotalol)	Healthy	Healthy: history, exam, normal ECG, normal laboratory tests, no medications, negative pregnancy test, reliable contraception **Exclusion:** LQTS, TdP risk factors, concomitant medication use, fluoroquinolone hypersensitivity, unable to have moxifloxacin based on screening
Graff et al. (2009)	*n* = 986 Control = 917 LQT2 = 30 Sotalol = 39	29 ± 7 (control) 45 ± 14 (LQT2)	F: 146 (15%, control) 19 (2%, LQT2) 11 (1%, sotalol) M: 771 (78%, control) 11 (1%, LQT2) 28 (2.8%, sotalol)	Control (407 ± 18) LQT2 (483 ± 35) Sotalol (403 ± 15 to 459 ± 14)	Healthy: history, exam, no medications LQT2: confirmed hERG mutation **Exclusion:** poor Holter ECG tracings
Vaglio et al. (2008)	*n* = 112 Control = 38 LQT1 = 49 LQT2 = 25	27.5 ± 8.1 (control) 34.3 ± 10.2 (LQT1) 35.5 ± 9.4 (LQT2)	F: 11 (29%, control) 34 (71%, LQT1) 19 (76%, LQT2) M: 27 (71%, control) 15 (29%, LQT1) 6 (24%, LQT2)	Control (413 ± 17) LQT1 (493 ± 29) LQT2 (510 ± 41)	Healthy: nonmutation carriers, normal QTc KCNH2 and KvLQT1 gene positive (26 LQT1 and 19 LQT2 families)
Kanters et al. (2004)	*n* = 50 Control = 13 hERG = 24 KvLQT1 = 13	>14	F: 9 (control) 16 (hERG) 8 (KvLQT1) M: 4 (control) 8 (hERG) 5 (KvLQT1)	Control (healthy) (378 ± 11) hERG (498 ± 13) KvLQT1 (479 ± 13)	Danish LQTS Clinic hERG or KvLQT1 genotype positive Control: healthy, unaffected (genotype negative from same families) Age > 14 years Artifact‐free ECG
Moss et al. (1995)	*n* = 153 Six LQTS families U = 77 A = 76 Chromosome 3 (*n* = 47) U = 28 A = 19 Chromosome 7 (*n* = 30) U = 13 A = 17 Chromosome 11 (*n* = 76) U = 36 A = 40	Chromosome 3: Family 1: 20 ± 17 (U) 27 ± 20 (A) Family 2: 30 ± 19 (U) 19 ± 13 (A) Chromosome 7: Family 3: 35 ± 23 (U) 27 ± 17(A) Family 4: 29 ± 13 (U) 29 ± 24 (A) Chromosome 11: Family 5: 19 ± 19 (U) 15 ± 12 (A) Family 6: 24 ± 23 (U) 27 ± 25 (A)	Chromosome 3: (F/M) Family 1: 9/13 (U) 3/10 (A) Family 2: 4/2 (U) 2/4 (A) Chromosome 7: (F/M) Family 3: 1/2 (U) 4/1 (A) Family 4: 6/4 (U) 6/6 (A) Chromosome 11: (F/M) Family 5: 2/5 (U) 4/3 (A) Family 6: 17/12 (U) 23/10 (A)	Chromosome 3: Family 1: 417 ± 35 (U) 535 ± 46(A) Family 2: 420 ± 30 (U) 523 ± 40 (A) Chromosome 7: Family 3: 407 ± 12 (U) 502 ± 49 (A) Family 4: 410 ± 35 (U) 458 ± 51 (A) Chromosome 11: Family 5: 417 ± 49 (U) 514 ± 44 (A) Family 6: 416 ± 36 (U) 491 ± 43 (A)	LQTS family Genotype positive Control: healthy, genotype negative from same families **Exclusion:** congenital hearing loss, left cervicothoracic sympathetic ganglionectomy

Abbreviations: A, affected; AF, atrial fibrillation; aLQTS, acquired long QT syndrome; BBB, bundle branch block; BMI, body mass index; BrS, Brugada syndrome; cLQTS, congenital long QT syndrome; ECG, electrocardiogram; F, female; LQT1, long QT syndrome type 1; LQT2, long QT syndrome type 2; LQTS, long QT syndrome; LVH, left ventricular hypertrophy; M, male; QTc, corrected QT interval; ST‐T, ST‐T segment; SVT, supraventricular tachycardia; TdP, torsades de pointes; TW, T wave; TWM, T wave morphology; U, unaffected; VE, ventricular ectopy; VT, ventricular tachycardia.

**TABLE 2:** Summary of results for congenital long QT syndrome.

Citation	Medication(s) & drug monitoring	ECG recording, QTc correction & analysis	Outcome measure	Results
Hermans et al. (2020)	N/A	12 lead ECG Bazett formula Automated ECG analysis software (MATLAB)	TW characterization: Hermite‐Gauss polynomials STT segment (1st order polynomials) Tpeak (2nd order polynomials) Machine learning: QTc cutoffs: >480 ms >450/460 ms (M/F) 99th percentile healthy subjects Models: Baseline (age, sex, QTc) Morphology (c_1_, c_2_, fitting error, age, sex) Extended (baseline and morphology features) QT expert: Cardiologist review ROC analysis (AUC, Sn, Sp) Youden's index Delong method (ROC comparison)	Extended model: 84% cLQTS classified correctly (QTc 400–460 ms) Accuracy: 84% (Amsterdam), 79% (Leuven) AUC > baseline>morphology models (*p* < .001) Sn: 83% (Amsterdam), 75% (Leuven) Enhanced performance vs. QTc cutoffs QTc cutoffs: Accuracy: ≥480 ms: 62% (Amsterdam), 52% (Leuven) >450/460 ms: 72% (Amsterdam), 65% (Leuven) Sp: 99% (Amsterdam), 98% (Leuven) Sn: 24% (Amsterdam), 24% (Leuven) LQTS subtypes: Highest accuracy for LQT3 (extended model) QT expert: Accuracy: 79% (Leuven) Sn: 75% (Leuven) Sp: 86% (Leuven) Extended model comparison: 87% agreement
Hermans et al. (2018)	N/A	12 lead ECG Bazett, Fridericia, Framingham, Hodges formulae Automated ECG analysis software (MATLAB)	Baseline model: Age, sex, RR‐interval, QT‐interval, QTc Extended model: Baseline model features TWM features Support network machine: Elastic net regularization (lasso and ridge regression) Mixing and tuning parameter (ROC analysis) Performance analysis: Maximal Youden's index (YI_max_) *= Sn + Sp ‐ 1*	AUC: Baseline model: 0.821 Extended model: 0.901 Maximal Youden's index: Baseline model: Sn 0.694, Sp 0.829 Extended model: Sn 0.820, Sp 0.861 QTc‐interval cut‐off values: Extended model: Reduction in false negative LQTS classification
Platonov et al. (2018)	N/A	12 lead ECG Bazett formula Cardiologist review	TWM: Flat, notched, negative (leads II, V_5_) Risk of cardiac events: Syncope, aborted cardiac arrest, defibrillator therapy, SCD	Distribution of abnormal TWM: Control: 55 LQT2: 64 Higher risk of cardiac events: Females with abnormal TWM (HR, 3.31; 95% CI, 1.68–6.52; *p* = .001) LQT2 men with pore‐located mutations vs. nonpore (HR, 6.01; 95% CI, 1.50–24.08; *p* = .011)
Porta‐Sanchez et al. (2017)	N/A	12 lead ECG Bazett formula QT Guard Plus software	MCS *= 1.6 x flatness + asymmetry + notch* PCA‐2	TWM‐specific differentiation: LQTS vs. control MCS: 117.8 ± 57.4 vs. 71.9 ± 16.2; *p* < .001; PCA‐2: 20.2 + 10.4% vs. 14.6 + 5.5%; *p* < .001 LQT1 vs. LQT2 MCS:96.3 ± 28.7 vs. 164 ± 75.2; *p* < .001; PCA‐2: 17.8 ± 8.3% vs. 25 ± 12.6%; *p* < .001 LQTS with normal QTc MCS: 105.7 ± 49.9 vs. 71.9 ± 16.2; *p* < .001, PCA‐2: 18.1 ± 7.2% vs. 14.6 ± 5.5%; *p* < .001
Sugrue, Rohatgl, et al. (2017)	N/A	12 lead ECG Bazett formula Automated QT‐alert system (QTc ≥500 ms) Cardiologist review	Novel proprietary TW program LQTS‐related BCE: Arrhythmogenic syncope, seizure, aborted cardiac arrest, appropriate ICD shock, SCD Beta‐blocker therapy analysis	BCE (≥1): *n* = 23 BCE risk factors: Lead V_6_: Left slope TW (HR = 0.40 [0.24–0.69]; *p* < .001), Lead I: TW COG x axis last 25% (HR = 1.90 [1.21–2.99]); *p* = .005, C statistic 0.77 (0.65–0.89) QTc increased discrimination to 0.78 (C statistic 0.68) TW variables>QTc in LQT2 (C statistic 0.82 [0.71–0.93]) Beta‐blocker therapy: similar risk
Immanuel et al. (2016)	N/A	Holter ECG (THEW) Automated signal processing toolbox (MATLAB)	Frequency binned averaged ECGs and TW extraction (lead I) Boltzmann sigmoidal functions (upslope, downslope, switch) 9th order polynomial functions (upslope, downslope) Neural network classifiers Normal QTc subgroup analysis (400–450 ms)	TW parameters: Sigmoidal and polynomial classifiers LQTS subtypes>conventional parameters (*p* < .0001) No difference control vs. LQTS (*p* = .19) or LQT1 vs. LQT2 Neural network classifiers: Control vs. LQTS: 92% LQT1 vs. LQT2: 88% Normal QTc subgroup analysis: TWM: 90% QTC: 71%
Sugrue et al. (2016)	N/A	12 lead ECG Bazett formula Automated TW analysis software (MATLAB)	Novel proprietary TW program Training and validation phases Manifest and concealed LQTS TW features vs. QTc Diagnostic accuracy: Sn, Sp, NPV, PPV	Discrimination accuracy TWM Lead V_6_: Manifest LQTS: 86.8% Concealed LQTS: 83.3% TW features: Tpeak‐Tend, left slope, COG *x* axis Sn: 83% (manifest), 76% (concealed) Sp: 91% (manifest), 91% (concealed) PPV: 90% (manifest), 89% (concealed) NPV: 84% (manifest), 79% (concealed)
Vaglio et al. (2008)	N/A	12 lead Holter ECG Bazett and Fridericia formulae Automated custom‐made algorithms	Scalar and vectorial repolarization parameters: QTpeak, Tpeak‐Tend interval, TW magnitude, T‐loop morphology, alpha‐R (right slope), alpha‐L (left slope) RI (derived from Hill equation) RI(*t*) *= V*_max_ × (*t* ^*n*^/[*k*_*m*_^*n*^ *+ t* ^*n*^]) *V*_max_: total TW area K_m_: time when 50% TW area reached *n*: slope of the sigmoid RI	Discriminatory TWM biomarkers: QTpeak (lead II) Alpha‐R (Tpeak‐Tend) H‐R effect: H‐R bin 75–77.5 bpm: best discrimination H‐R range 60–100 bpm: 89% control, 84% LQT1, 92% LQT2 Bradycardia>tachycardia Scalar model: RR, QTpeak, Tpeak‐Tend, TW magnitude 90% discrimination with automated computer system (89% control, 90% LQT1, 92% LQT2) Vectorial model: Alpha‐R (vectorgraphic leads), QTpeak (lead II) 90% discrimination with automated computer system (92% control, 88% LQT1, 91% LQT2) RI: H‐R range 75 to 77.5 bpm Amplitude and morphology for LQT2 < LQT1 *n* (*p* < .001) *V*_max_ (*p* < .018)
Kanters et al. (2004)	N/A	12 lead ECG Bazett formula UN‐SCAN‐IT software (ECG digitization) Microcal Origin software (ECG fitting)	TWM parameters (lead II, V_2_, V_5_) RI (derived from Hill equation) RI(*t*) *= V*_max_ *× (t* ^*n*^ */[k*_*m*_^*n*^ *+ t* ^*n*^]) *V*_max_: total TW area *K*_*m*_: time when 50% TW area reached *n*: slope of the sigmoid RI	RI: correlated to fitted sigmoid (*r* = .99) *V*_max_: largest in KvLQT1 Leads V_2_, V_5_, II: *n* of RI discriminates hERG from KvLQT1 Sn and Sp 100% (*p* < .05)
Moss et al. (1995)	N/A	12 lead ECG Bazett formula	ECG leads: II, aVF, V_5_ ECG biomarkers: RR interval, QTc‐onset, QTc‐peak, QTc, T‐duration, T‐amplitude	QT intervals: QTc, QTc‐onset, QTc‐peak prolonged in affected (*p* < .01) QTc‐onset: longest chromosome 3 (*p* < .001) T‐amplitude: reduced chromosome 7 (*p* < .001) T‐duration: longest chromosome 11 (*p* < .001) Lead correlation: aVF and V_5_ equivalent

Abbreviations: AUC, area under the curve; BCE, breakthrough cardiac events; bpm, beats per minute; CI, confidence interval; COG, center of gravity; ECG, electrocardiogram; H‐R, heart rate; HR, hazard ratio; hr, hour; ICD, implantable cardioverter defibrillator; LQTS, long QT syndrome; MCS, morphology combination score; NPV, negative predictive value; PCA‐2, principal component analysis ratio 2; PPV, positive predictive value; RI, repolarizing integral; ROC, receiver‐operating curve; RR, R‐R interval; SCD, sudden cardiac death; Sp, specificity; Sn, sensitivity; THEW, Telemetric and Holter ECG Warehouse database; TW, T wave; TWM, T wave morphology.

**TABLE 3:** Summary of results for acquired long QT syndrome.

Citation	Medication(s) and drug monitoring	ECG recording, QTc correction and analysis	Outcome measure	Results
Sugrue, Noseworthy, et al. (2017)	N/A	12 lead ECG Bazett formula Automated QT‐alert system (QTc ≥500 ms) Cardiologist review	Novel proprietary TW program: TW left/right slope, TW amplitude, TW area, TW COG *x/y* coordinates, COG first 25% TW, COG last 25% of the TW, Tpeak–Tend interval Diagnostic accuracy: Sn, Sp, NPV, PPV Sensitivity analysis (age, sex)	aLQTS TWM biomarkers: Lead V_5_ Shallower TW right slope (−2322 vs. −3593 mV/s; *p* < .001) Greater T‐peak‐Tend (109 vs. 92 ms; *p* < .001) Smaller TW COG (290 ms vs. 310 ms; *p* < .001) cLQTS vs. aLQTS: Lead V_5_ distinguishes cLQTS from aLQTS in 77% cases Sn 90%, Sp 58%, PPV 83%, NPV 71% Sensitivity analysis: Age 78.3% No sex difference (M: 76%, F: 78%)
Sugrue et al. (2015)	Sotalol Dofetilide	12 lead ECG Bazett formula Automated TW analysis software (MATLAB)	Novel proprietary TW program QT interval and QTc Pre and post TdP ECG Risk prediction: Sn, Sp, NPV, PPV	Discrimination accuracy Lead V_3_: QTc discrimination (*p* < .001, *r* = .72) Drug: 480 ms Control: 420 ms Lead I: TW right slope (*p* = .002, *r* = .45) Torsadogenic risk prediction: ECG biomarkers TWRS: 88% QTc alone: 79% Risk prediction accuracy Sn: 92.1% (combined), 88.1% (QTc), 79.7% (TWRS) Sp: 81.4% (combined), 72% (QTc), 46% (TWRS) PPV: 90.3% (combined), 85% (QTc), 58.9% (TWRS) NPV: 84.6% (combined), 76.9% (QTc), 70% (TWRS)
Vicente et al. (2015)	Dofetilide (500 mcg) Quinidine (400 mg) Ranolazine (1500 mg) Verapamil (120 mg) 24 hr treatment period 7 day washout period Plasma drug level paired with ECG	12 lead ECG (THEW): Baseline 15 time‐points postdose (0.5, 1, 1.5, 2, 2.5, 3, 3.5, 4, 5, 6, 7, 8, 12, 14, 24 hr) Fridericia formula QTGuard+ automated TWM and vectorcardiographic biomarker analysis	ECG biomarkers: QT interval, QRS, J‐Tpeak interval, Tpeak–Tend interval TWM architectural patterns: Flatness, asymmetry, notching Vectorcardiographic biomarkers: ERD_30%_, LRD_30%_, QRS‐T angle, ventricular gradient, maximum magnitude of T vector, TCRT Patch clamp experiments: Ion channel block (hERG, late sodium, calcium)	Ion channel block: Dofetilide: 55% hERG Quinidine: 71% hERG, 8% calcium, 3% sodium Ranolazine: 26% hERG, 21% sodium Verapamil: 17% calcium, 7% hERG ΔQTc & TWM metrics: Dofetilide: ΔQTc 73.6 ms, flatness 0.16, asymmetry 0.25, notching 55% (*p* < .001) Quinidine: ΔQTc 78.9 ms, flatness 0.21, asymmetry 0.34 (*p* < .01), notching 69.7% (*p* < .001) Ranolazine: ΔQTc 12 ms, flatness 0.06 (*p* < .01), asymmetry 0.10, notching 1.4% (*p* < .001) Vectorcardiographic biomarkers: ERD_30%_, LRD_30%_ increase for dofetilide, quinidine, ranolazine QRS‐T angle decrease for dofetilide, quinidine TCRT decrease for dofetilide
Johannessen et al. (2014)	Dofetilide (500 mcg) Quinidine (400 mg) Ranolazine (1500 mg) Verapamil (120 mg) 24‐hr treatment period 7 ‐day washout period Plasma drug level paired with ECG	12 lead ECG (THEW) Fridericia formula QTGuard+	ECG biomarkers (lead II): P‐onset, PR, QRS, QRS‐onset, QRS‐offset, QT interval, J‐Tpeak interval, Tpeak–Tend interval Concentration‐dependent analysis	QTc & TWM metric prolongation: Dofetilide: QTc 79.3 ms, J‐Tpeak 39.5 ms, Tpeak–Tend 40 ms, CDA; (*p* < .001) Quinidine: QTc 78.1 ms, J‐Tpeak 29.1 ms, Tpeak–Tend 49.8 ms, CDA; (*p* < .001) Ranolazine: QTc 12.6 ms (*p* < .001), Tpeak–Tend 8.8 ms (*p* < .013), CDA (*p* < .01)
Couderc et al. (2011)	Moxifloxacin ECG (2 hr post)	12 lead ECG Bazett and Fridericia formulae ECGScan software for ECG digitization Automated ECG interval assessment with COMPAS software	Cardiac ventricular repolarization: Lead II: QT apex, QT, Tpeak‐Tend, TW amplitude, left and right TW slope Eigen lead: T‐loop (roundness) Vectorcardiographic biomarkers: ERD, LRD (30–50% threshold) Cardiac events	Moxifloxacin model: ERD positive with drug (*p* = .0001) LQT2 model: Left slope detected KCNH2 mutation (*p* = .0002) Cardiac event prediction: T‐roundness (*p* = .007) Equivalent to QTc
Graff et al. (2010)	Trial 1 Placebo: 7 days Moxifloxacin Days 1–6: placebo Day 7: 400 mg Trial 2 Sotalol Day 1: no drug Day 2: 160 mg Day 3: 320 mg	Trial 1 12 lead ECG postdose Trial 2 Digital Holter ECG Fridericia formula MUSE/Interval Editor software	MCS *= asymmetry + notch + (1.6 x flatness)* QTc	Sotalol MCS > QTc (z score 2.5, CI 1.26 to 3.66) 320 mg equivalent to 160 mg (*p* = .25) 160 mg: MCS 7x greater, QTc 4x greater (vs. moxifloxacin) 320 mg: MCS 15x greater, QTc 6x greater (vs. moxifloxacin) Covariate effects on QTc and TWM: Parabolic regression>linear regression for sotalol (*p* < .01) No difference for moxifloxacin (*p* = .45) ΔMCS: Sotalol 320 mg > 160 mg (pooled fit, *p* < .01) Sotalol 160 mg > moxifloxacin and placebo Moxifloxacin sex differences QTc and TWMΔ: F > M
Graff et al. (2009)	Sotalol Day 1: 0 mg Day 2: 160 mg Day 3: 320 mg Plasma drug level	12 lead ECG Control LQT2 12 lead Holter ECG Sotalol Fridericia formula 12SL algorithm	MCS *= asymmetry + notch + (1.6 x flatness)* QTc Test accuracy (AUC)	Sotalol: MCS > QTc (*p* < .001) 160 mg (AUC 84% vs. 72%) 320 mg (AUC 94% vs. 87%) Effect size: MCS > QTc (50%) at maximal AUC and ECGΔ (*p* < .001) MCS > QTc (3‐fold) at peak ECGΔ (*p* < .001) MCS discrimination accuracy (normal QTc) Similar for sotalol and LQT2 (*p* = .9)

Abbreviations: Δ, delta change; AUC, area under the curve; CI, confidence interval; COG, center of gravity; COMPAS, COMPrehensive Analysis of the repolarization Segment; ECG, electrocardiogram; ERD, early repolarisation; F, female; hr, hour; LRD, late repolarization; LQTS, long QT syndrome; M, male; MCS, morphology combination score; NPV, negative predictive value; PPV, positive predictive value; Sp, specificity; Sn, sensitivity; TCRT, total cosine R‐to‐T; THEW, Telemetric and Holter ECG Warehouse database; TW, T wave; TWM, T wave morphology; TWRS, T wave right slope.

### 3.2 Cohort characteristics

Table 1 summarizes the key cohort characteristics. A total of 5925 subjects were enrolled across 17 studies, ranging in size from 22 to 1161 subjects. The same cohort was utilized for different studies in two instances. This included 22 subjects in two studies (Johannessen et al., 2014; Vicente et al., 2015) and 678 subjects across another two studies (Hermans et al., 2018; Hermans et al., 2020), with an additional 10 subjects meeting the inclusion criteria in Hermans et al. (2020).

Of the studies which specified sex differences, 2462 females and 2481 males were included. Of the remaining studies approximately two‐thirds of subjects were female and one‐third were male, lending to a female predominance overall (Porta‐Sanchez et al., 2017; Sugrue et al., 2015).

Age data ranged from a mean of 16 (Sugrue, Rohatgl, et al., 2017) to 68.4 ± 5.5 (Sugrue et al., 2015) years, reflective of cLQTS and aLQTS cohorts, respectively. A single study did not outline the age of subjects, however their inclusion criteria was greater than 14 years (Kanters et al., 2004).

#### 3.2.1 Health status and LQTS type

Health status of the subjects included healthy individuals based on clinical assessment (Couderc et al., 2011; Graff et al., 2009; Graff et al., 2010; Immanuel et al., 2016; Platonov et al., 2018; Porta‐Sanchez et al., 2017; Sugrue et al., 2016), genotype negative individuals in LQTS affected families (Hermans et al., 2018; Hermans et al., 2020; Kanters et al., 2004; Moss et al., 1995; Vaglio et al., 2008), LQT1 (Hermans et al., 2018; Hermans et al., 2020; Immanuel et al., 2016; Kanters et al., 2004; Porta‐Sanchez et al., 2017; Sugrue et al., 2016; Sugrue, Noseworthy, et al., 2017; Sugrue, Rohatgl, et al., 2017; Vaglio et al., 2008), LQT2 (Couderc et al., 2011; Graff et al., 2009; Hermans et al., 2018; Hermans et al., 2020; Immanuel et al., 2016; Johannessen et al., 2014; Kanters et al., 2004; Platonov et al., 2018; Porta‐Sanchez et al., 2017; Sugrue et al., 2016; Sugrue, Noseworthy, et al., 2017; Sugrue, Rohatgl, et al., 2017; Vaglio et al., 2008; Vicente et al., 2015), LQT3 (Hermans et al., 2018; Hermans et al., 2020), hERG or KCNH2 and KvLQT1 mutations (Kanters et al., 2004; Vaglio et al., 2008); and chromosome‐specific mutations affecting chromosomes 3 (*SCN5A*), 7 (*KCNH2*) and 11 (*KCNQ1*; Moss et al., 1995). Among healthy subjects, aLQTS was identified retrospectively in the presence of QTc prolonging medications and electrolyte derangement (Sugrue, Noseworthy, et al., 2017), and following admission for commencement of sotalol or dofetilide antecedent to documented TdP (Sugrue et al., 2015). Two studies assessed the effect of multichannel blockade using dofetilide (500 mcg), quinidine (400 mg), ranolazine (1500 mg), and verapamil (120 mg) on repolarization ECG biomarkers in a healthy cohort (Johannessen et al., 2014; Vicente et al., 2015). Similarly, administration of moxifloxacin (Couderc et al., 2011; Graff et al., 2010) and sotalol (Graff et al., 2009; Graff et al., 2010) in healthy individuals was undertaken to compare ECG changes in iatrogenic aLQTS and LQT2.

#### 3.2.2 ECG recording and QT interval correction

Electrocardiograms (ECGs) were recorded at rest in the supine position (Couderc et al., 2011; Graff et al., 2009; Graff et al., 2010; Hermans et al., 2018; Hermans et al., 2020; Johannessen et al., 2014; Kanters et al., 2004; Moss et al., 1995; Platonov et al., 2018; Porta‐Sanchez et al., 2017; Sugrue et al., 2015; Sugrue et al., 2016; Sugrue, Noseworthy, et al., 2017; Sugrue, Rohatgl, et al., 2017; Vicente et al., 2015) or by Holter monitor (Graff et al., 2009; Graff et al., 2010;Immanuel et al., 2016; Vaglio et al., 2008). Of the studies which included data collected from Holter monitors, three (Graff et al., 2009; Graff et al., 2010; Vaglio et al., 2008) utilized 12 lead ECGs sampling at 180 Hz and one (Immanuel et al., 2016) used two or three lead ECGs sampling at 200 Hz. With regards to processing of digitized Holter ECGs, two studies (Graff et al., 2009; Graff et al., 2010) used the same methodology for heart rate normalization, beat binning, filtering; and fiducial point identification, whereas variations across these factors were noted for the other studies (Immanuel et al., 2016; Vaglio et al., 2008). Specific Holter ECG lead selection occurred in two studies to facilitate application of TW assessment techniques (lead I; Immanuel et al., 2016; leads II and V_5_; Vaglio et al., 2008).

QT correction formulae used included Bazett in 13 studies (Couderc et al., 2011; Hermans et al., 2018; Hermans et al., 2020; Immanuel et al., 2016; Kanters et al., 2004; Moss et al., 1995; Platonov et al., 2018; Porta‐Sanchez et al., 2017; Sugrue et al., 2015; Sugrue et al., 2016; Sugrue, Noseworthy, et al., 2017; Sugrue, Rohatgl, et al., 2017; Vaglio et al., 2008), the Fridericia in seven studies (Couderc et al., 2011; Graff et al., 2009; Graff et al., 2010; Hermans et al., 2018; Johannessen et al., 2014; Vaglio et al., 2008; Vicente et al., 2015), and Framingham and Hodges in one study (Hermans et al., 2018).

#### 3.2.3 T wave‐specific ECG measurements

**FIGURE 2:**
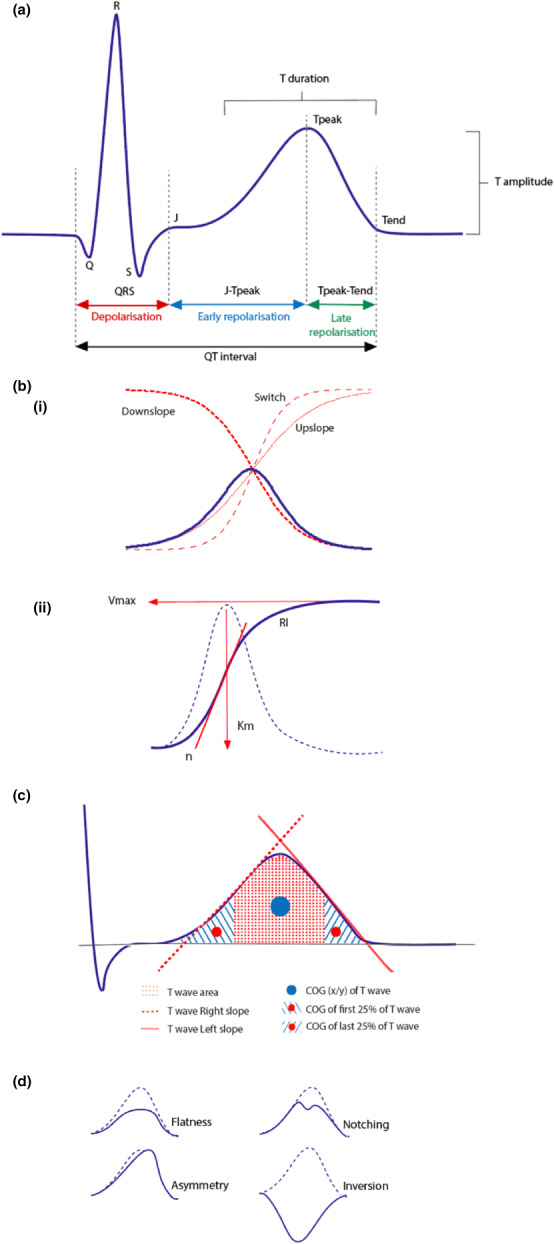
Selected T wave morphology analysis techniques. (a) ECG time intervals indicating specific T wave parameters, including Tpeak‐Tend interval (ms), T duration (ms), and T amplitude (mV). (bi) Application of sigmoidal classifiers demonstrated using Boltzmann sigmoidal functions: Upslope (red dotted line), downslope (red bold dotted line), and switch (red dashed line), as adapted from Immanuel et al. (2016). (bii) T wave fitting of the repolarizing integral (RI), derived from three Hill parameters: *n* (red bold slope), *V*_max_ (red horizontal arrow), *K*_*m*_ (red vertical arrow) as adapted from Kanters et al. (2004). (c) T wave features applied by the novel, *proprietary T wave program*, including T wave area, T wave right and left (mV/s), COG (*x*/*y*) of T wave; and COG of first and last 25% of T wave (ms) as adapted from Sugrue et al. (2016). (d) T wave architectural patterns

In addition to measurement of QT intervals, three other sets of features have been extracted from the ECG to help distinguish LQTS subjects from controls. These include (i) additional time intervals (Figure 2a), (ii) TWM markers (Figure 2b,d), and (iii) vectorcardiography‐derived measurements (Figure 2c), with most studies employing multiple TWM measurements. Tables 2 and 3 summarize these methods and their application across studies.

Briefly, TWM biomarkers comprised of assessment of TW architecture visually (Figure 2d; Immanuel et al., 2016; Platonov et al., 2018; Vicente et al., 2015) or via use of the principal component analysis (PCA; Hermans et al., 2018; Porta‐Sanchez et al., 2017; Vicente et al., 2015) as described by Anderson et al. (Anderson et al., 2007), sigmoidal and polynomial functions (Hermans et al., 2020; Immanuel et al., 2016), TW area measured by the repolarizing integral (RI; Figure 2bii) as derived from the Hill equation (Table 2; Kanters et al., 2004; Vaglio et al., 2008), *proprietary T wave program* (Figure 2c; Sugrue et al., 2015; Sugrue et al., 2016; Sugrue, Noseworthy, et al., 2017; Sugrue, Rohatgl, et al., 2017); and morphology combination score (MCS; Tables 2 and 3; Graff et al., 2009; Graff et al., 2010; Porta‐Sanchez et al., 2017).

**FIGURE 3:**
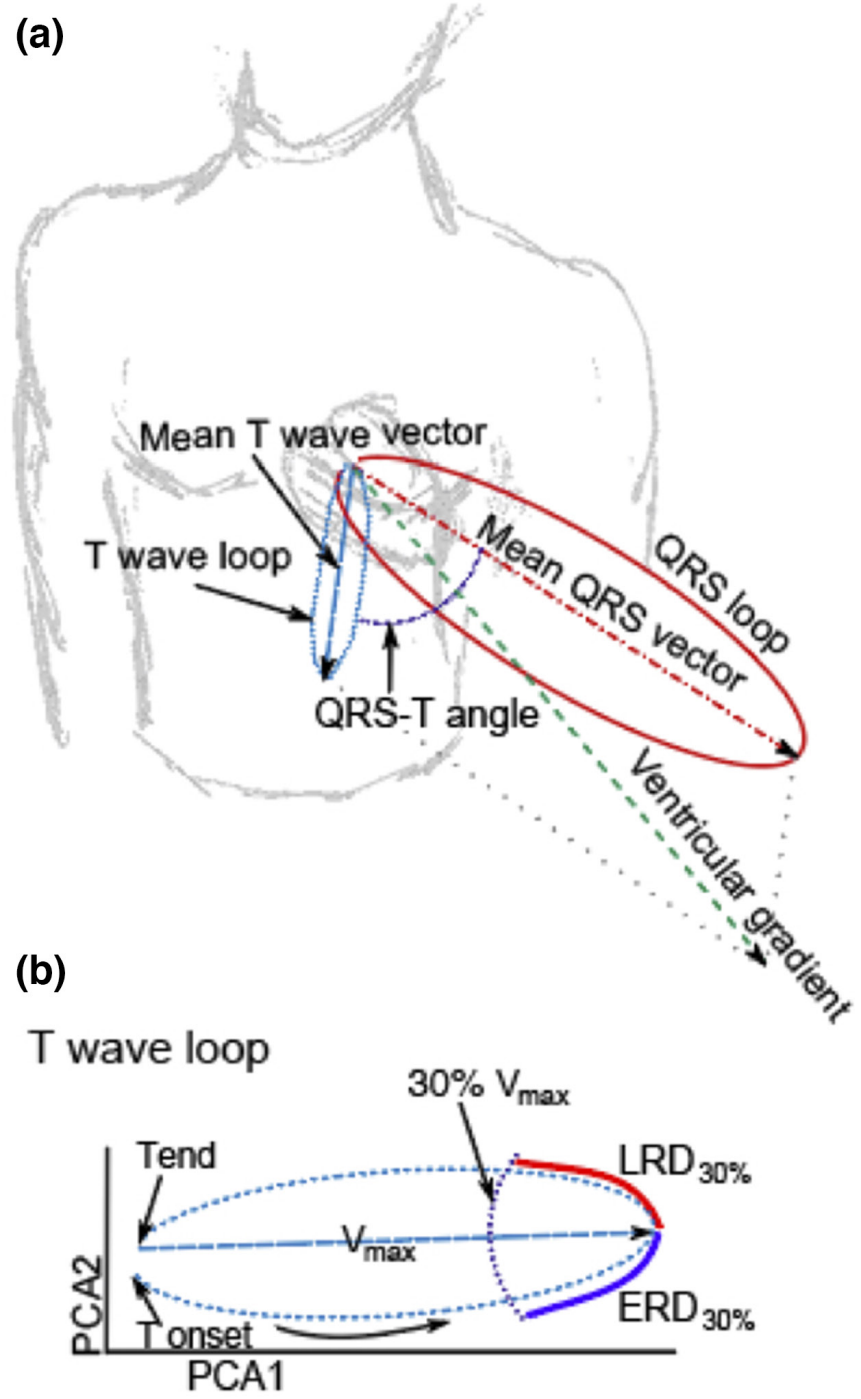
Vectorcardiographic biomarkers including (a) QRS‐T angle (dark blue dotted line), ventricular gradient (green dashed line), maximum magnitude of the T vector (derived from the QRS loop [dark red solid line], and T wave loop [light blue dashed line]); (b) and 30% early (ERD_30%_, blue solid line) and late (LRD_30%_, red solid line) repolarization of the T wave loop as adapted from Vicente et al. (2015).

The key vectorcardiographic parameters utilized were defined by Vicente et al. (2015) as follows:

- *QRS‐T angle*: the angle between the QRS and T vectors, which were described as the summation in X, Y, and Z leads from the QRS onset to QRS offset to T offset, respectively (Figure 3a)
- *Ventricular gradient*: magnitude of the sum of the QRS and T vectors (Figure 3a)
- *Maximum T vector*: the vector with the maximum magnitude between QRS offset and T offset
- *Total cosine R‐to‐T (TCRT)*: concordance between ventricular depolarization and repolarization sequences
- *Early (ERD) and late (LRD) repolarization*: time from the peak of the TW loop to 30% of the baseline toward the beginning of the TW (ERD_30%_) and end of the TW (LRD_30%_; Figure 3b).

### 3.3 Utility of different ECG parameters for distinguishing between controls and cLQTS

#### 3.3.1 QT parameters

Hermans et al. (2020) demonstrated QTc cutoffs >480 ms had the poorest performance with regards to diagnostic accuracy for both the study cohort (Amsterdam data) and external cohort (Leuven data), that being 62% and 52%, respectively, in individuals with LQTS (LQT1, LQT2, LQT3) compared to genotype negative family member controls. Some studies have investigated whether the QTpeak might be more useful. For example, QTpeak in lead II, has been used to differentiate control from LQT1 and LQT2 (Vaglio et al., 2008).

#### 3.3.2 T wave parameters

Moss et al. (1995) noted prolongation of TW duration in lead II for genotype positive individuals with mutations of chromosome 11 (262 ± 65 ms) compared to chromosome 3 (187 ± 33 ms) and chromosome 7 (0.37 ± 0.17 ms; *p* < .001). A reduction in TW amplitude in lead II was seen in chromosome 7 mutations (0.13 ± 0.07 millivolts [mV]) compared to chromosome 3 (0.36 ± 0.14 mV) and chromosome 11 (0.37 ± 0.17 mV; *p* < .001).

Assessment of three combined scalar ECG parameters, including Tpeak‐Tend, TW magnitude, and QTpeak, achieved ~90% discrimination across control, LQT1 and LQT2 (Vaglio et al., 2008). Left and right slope were shown in separate studies to detect the KCNH2 mutation in an LQT2 model (left tangent in lead II, odds ratio [OR] 0.38, 95% confidence interval [CI]: 0.23–0.64; *p* = .0002; Couderc et al., 2011) and discriminate control from LQT1 and LQT2 (best vectorcardiographic parameter correctly identifying 69% of individuals; Vaglio et al., 2008), respectively. Lead V_6_ discriminated between LQTS (86.8%) and concealed LQTS (83.3%) compared to control, based on Tpeak‐Tend, left slope; and COG *x* axis (Sugrue et al., 2016). Lead V_5_ discriminated aLQTS from cLQTS based on shallower right slope (−2322 vs. −3593 mV/s), longer Tpeak‐Tend (109 vs. 92 ms) and smaller COG (290 vs. 310 ms; *p* < .001; Sugrue, Noseworthy, et al., 2017).

#### 3.3.3 Sigmoidal and polynomial classifiers

Sigmoidal and polynomial classifiers were more significant than conventional parameters, including Q‐Tend tangent, Tpeak‐Tend; and height of Tpeak, for distinguishing cLQTS subtypes (*p* < .0001; Immanuel et al., 2016). Immanuel et al. (2016) also used a neural network classifier (NNC) approach to investigate whether extracted TWM markers could improve diagnostic classification of cLQTS versus control, and cLQTS subtypes. NNCs demonstrated an ability to discriminate between control versus LQTS, and LQT1 versus LQT2, at 92% and 88%, respectively (Immanuel et al., 2016). Application of this approach in the subgroup with a normal QTc showed the sigmoidal and polynomial classifiers were better than QTc alone for discriminating between controls and cLQTS, and between LQTS1 and LQTS2, at rates of 90% and 70%, respectively (Immanuel et al., 2016).

Machine learning was applied by Hermans et al. (2020) using “baseline,” “morphology,” and “extended” support vector machine (SVM) models. The “extended” SVM model added TWM parameters, including Hermite‐Gauss polynomials, to those included in the “baseline” model (age, sex, and QTc), to determine their impact on differentiating cLQTS from gene negative relative controls at various QTc cutoffs. Addition of Hermite‐Gauss polynomials improved diagnostic accuracy of cLQTS individuals compared with controls, as demonstrated by the area under the curve (AUC) of receiver operator characteristic (ROC) curves (“extended” model AUC 0.90 [0.88–0.93]) versus “morphology” (AUC 0.81 [0.77–0.84]) and “baseline” (AUC 0.87 [0.84–0.90]) models (*p* < .001; Hermans et al., 2020). The same SVM model achieved correct classification in 84% of genotype positive cLQTS patients with a normal QTc, ranging between 400 and 460 ms (Hermans et al., 2020).

#### 3.3.4 Repolarizing integral

RI correlated with sigmoidal function fitting of the TW (*r* = .99), with the slope of the RI sigmoid differentiating between hERG and KvLQT1 mutations in leads V_2_, V_5,_ and II, with a sensitivity and specificity of 100% (*p* < .05; Kanters et al., 2004). RI characteristics of *n* and *V*_max_, measures characterizing amplitude and morphology, were also shown to differentiate between cLQTS subtypes (Vaglio et al., 2008). Specifically, within a heart rate range of 75 to 77.5 beats per minute *n* and *V*_max_ were both found to be reduced for LQT2 compared to LQT1 at a significance of *p* < .001 (*n*: 5.3 ± 2.9 in LQT1 and 4.6 ± 3.8 in LQT2) and *p* < .018 (*V*_max_: 0.16 ± 0.07 mV s in LQT1 and 0.10 ± 0.05 mV s in LQT2), respectively (Vaglio et al., 2008).

#### 3.3.5 Morphology combination score and principal component analysis

PCA‐2 and the subsequent MCS differentiated cLQTS from control (MCS: 117.8 ± 57.4 vs. 71.9 ± 16.2; *p* < .001; PCA‐2: 20.2 ± 10.4% vs. 14.6 ± 5.5%; *p* < .001), LQT1 from LQT2 (MCS: 96.3 ± 28.7 vs. 164 ± 75.2; *p* < .001: PCA‐2: 17.8 ± 8.3% vs. 25 ± 12.6%; *p* < .001) and cLQTS with normal QTc (MCS: 105.7 ± 49.9 vs. 71.9 ± 16.2; *p* < .001; PCA‐2: 18.1 ± 7.2% vs. 14.6 ± 5.5%; *p* < .001; Porta‐Sanchez et al., 2017). ROC curves were used to assess diagnostic performance of PCA‐2 and MCS, demonstrating superiority for MCS with a sensitivity of 79%, specificity of 82.6%, and global accuracy of 80.6% (ROC area 0.88) versus sensitivity of 59.7%, specificity of 60.9% and global diagnostic accuracy of 60.2% (ROC area 0.69) for PCA‐2; *p* = .002 (Porta‐Sanchez et al., 2017).

#### 3.3.6 Vectorcardiographic biomarkers

Incorporation of spatial peak QRS‐T angle (smallest angle between the vector at maximal TW magnitude and the vector at maximal QRS) and spatial mean QRS‐T angle (smallest angle between the mean vector of the TW and the mean vector of the QRS) to the TWM features used in the SVM “extended” model, in addition to the “baseline” model coefficients of age, sex, RR‐interval, QT‐interval and QTc, improved AUC, sensitivity and specificity in cLQTS individuals compared with genotype negative family members (Hermans et al., 2018).

Vaglio et al. (2008) produced a computerized vectorial model by incorporating the vectorcardiographic biomarkers of right and left slopes and TW loop morphology, with fiducial points from their scalar model and parameters derived from the RI. The right slope in combination with QTpeak in lead II achieved discrimination rates of 92% for healthy, 88% for LQT1, and 91% for LQT2 subjects, respectively (Vaglio et al., 2008).

### 3.4 Utility of T wave morphology for identifying cLQTS

In cLQTS, TWM biomarkers were capable of delineating between genotype positive individuals, concealed LQTS with a normal QTc (Hermans et al., 2018; Hermans et al., 2020; Immanuel et al., 2016; Porta‐Sanchez et al., 2017), and controls (Hermans et al., 2018; Hermans et al., 2020; Immanuel et al., 2016; Porta‐Sanchez et al., 2017;Sugrue et al., 2016; Vaglio et al., 2008), differentiating between genotypes (Couderc et al., 2011; Hermans et al., 2018; Hermans et al., 2020; Kanters et al., 2004; Moss et al., 1995; Porta‐Sanchez et al., 2017; Vaglio et al., 2008); and were more efficacious at identifying cLQTS than the QTc (Graff et al., 2009; Hermans et al., 2018; Hermans et al., 2020). Relevant biomarkers identified included Tpeak‐Tend (Sugrue et al., 2016; Vaglio et al., 2008), left slope (Couderc et al., 2011; Sugrue et al., 2016), right slope (Vaglio et al., 2008), COG *x* axis (Sugrue et al., 2016), TW magnitude (Vaglio et al., 2008), QTpeak (Vaglio et al., 2008), T amplitude and T duration (Moss et al., 1995), T‐roundness (Couderc et al., 2011), MCS (Graff et al., 2009; Porta‐Sanchez et al., 2017), sigmoidal and polynomial classifiers (Hermans et al., 2020; Immanuel et al., 2016); and RI (Kanters et al., 2004).

Identification of cLQTS based on lead specificity was demonstrated for I (sigmoidal and polynomial classifiers; Immanuel et al., 2016), II (Q wave to Tpeak) (Vaglio et al., 2008), RI (Kanters et al., 2004), V_2_ (RI) (Kanters et al., 2004), V_5_ (RI; Kanters et al., 2004); and V_6_ (Tpeak‐Tend, left slope, COG *x* axis; Sugrue et al., 2016).

### 3.5 Utility of T wave morphology for identifying aLQTS

In aLQTS, TWM biomarkers discriminated aLQTS from cLQTS (Sugrue, Noseworthy, et al., 2017), correlated with QTc prolongation (Johannessen et al., 2014; Vicente et al., 2015), reflected specific pharmacological blockade (Couderc et al., 2011; Johannessen et al., 2014; Vicente et al., 2015); and were more proficient at identifying aLQTS than QTc (Graff et al., 2010).

QTc prolongation was associated with TWM flatness, asymmetry, and notching in multichannel block with dofetilide (*p* < .001), quinidine (*p* < .01 to *p* < .001), and ranolazine (*p* < .01 to *p* < .001; Vicente et al., 2015). In the same cohort, an independent analysis showed QTc prolongation was associated with prolonged J‐Tpeak for dofetilide (*p* < .001) and quinidine (*p* < .001), and Tpeak‐Tend for dofetilide (*p* < .001), quinidine (*p* < .001), and ranolazine (*p* < .013) which was significant on the basis of a concentration‐dependent analysis (dofetilide and quinidine *p* < .001, ranolazine *p* < .01; Johannessen et al., 2014).

MCS was more efficacious than QTc at adequate doses of sotalol (160 mg and 320 mg) compared to moxifloxacin and placebo (Graff et al., 2010). No difference was identified in a comparison of MCS between those taking sotalol and LQT2, independent of QTc (*p* = .9; Graff et al., 2009).

A model investigating multichannel block showed an increase in ERD_30%_ and LRD_30%_ for dofetilide, quinidine, and ranolazine, decrease in QRS‐T angle for dofetilide and quinidine; and decrease in TCRT for dofetilide (Vicente et al., 2015). ERD (30–50%) was shown to reflect hERG blockade in a moxifloxacin model following drug administration (*p* = .0001; Couderc et al., 2011).

Lead specificity was shown for determination of aLQTS in V_5_ based on shallower right slope, longer Tpeak‐Tend; and smaller COG (Sugrue, Noseworthy, et al., 2017).

### 3.6 Relationship of T wave morphology and risk stratification in cLQTS

TW abnormalities, identified as flatness, notching, and inversion in leads II and V_5_, were associated with higher risk of cardiac events in females (HR, 3.31; 95% CI, 1.68–6.52; *p* = .001) and males with pore‐located mutations (HR, 6.01; 95% CI, 1.50–24.08; *p* = .011), versus nonpore mutations, from a cohort with LQT2 (Platonov et al., 2018). Breakthrough cardiac events (BCE) were associated with TW left slope in lead V_6_ (*p* < .001) and COG last 25% in lead I (*p* = .005) in LQT1 and LQT2 (Sugrue, Rohatgl, et al., 2017). These variables were better predictors of BCE than QTc (C statistic 0.82 [0.71–0.93]), with risk found to be similar, independent of prophylactic beta‐blocker use (Sugrue, Rohatgl, et al., 2017).

### 3.7 Relationship of T wave morphology and risk stratification in aLQTS

In a group of patients admitted for initiation of sotalol or dofetilide, retrospective analysis of those who developed Torsades de Pointes, compared to those who did not, identified TW right slope in lead I (88%) as the best predictive marker (*p* = .002 compared to QTc alone; Sugrue et al., 2015).

In one study that compared cLQTS2 with aLQTS, roundness of the T‐wave, derived from the TW loop, was equivalent to the QTc in detecting cardiac events compared to those who remained event free (0.38 ± 0.17 vs. 0.47 ± 0.19, *p* = .007; Couderc et al., 2011).

## 4 DISCUSSION

TWM biomarkers are useful in identifying repolarization abnormalities in both cLQTS and aLQTS (Table 4). The clinical utility and suitability for risk stratification purposes of such measures is predominantly challenged by variations in study design and methodology.

**TABLE 4:** T wave morphology characteristics for common causes of congenital and acquired long QT syndrome.

	Typical T wave morphology characteristics
Congenital LQTS	
Long QT type 1 (KCNQ1)	Broad based
Long QT type 2 (KCNH2)	Bifid (notched), low voltage T wave alternans (biphasic)
Long QT type 3 (SCN5A)	Late onset (prolonged ST segment), high amplitude and narrow T wave
Calmodulin (CALM1,2,3)	T wave alternans
Triadin (TRDN)	Extensive T wave inversion (precordial leads)
Anderson‐Tawil syndrome (*KCNJ2*)	Broad based (prolonged T wave downslope) Bifid (wide T‐U junction)
Timothy syndrome (CACNA1c)	Late onset, small T waves Giant negative T waves (inversion) T wave alternans
Ankyrin‐B syndrome (ANK2)	Broad based T wave inversion Bifid
Acquired LQTS	
Hypocalcemia	T wave flattening, broad based
Hypokalemia	Bifid (U wave may be present) T wave flattening T wave inversion
Hypomagnesemia	T wave flattening, broad based
Hypothermia	Broad based Biphasic (T wave alternans)
Hypothyroidism	T wave inversion
Pheochromocytoma	Giant negative T waves (inversion)
Quinidine	T wave flattening Bifid, broad (U wave may be present)
Stroke	Deep T wave inversion
Takotsubo's cardiomyopathy	T wave inversion

ECG data were collected using resting 12 lead traces in most studies, with Holter monitors being utilized in four studies (Graff et al., 2009; Graff et al., 2010; Immanuel et al., 2016; Vaglio et al., 2008). Although Holter recordings are not yet standard in diagnosing LQTS, QTc, and morphology assessments using this method may facilitate diagnosis and genotype identification reinforcing their role in this setting (Mauriello et al., 2011; Vaglio et al., 2008; Waddell‐Smith et al., 2017). Benefits over 10 second resting ECGs include obtaining a richer source of dynamic data over 24 hours of continuous recording, analysis of rate‐dependence achieved using an extended selective beat binning approach, which accounts for inter‐beat variability and changes in heart rate (Hodkinson et al., 2016; Immanuel et al., 2016). Holter recordings, however, can be confounded by increased noise consequent to movement artifacts. Furthermore, Holters typically utilize relatively low digitization rates (125–200 Hz) compared to 500 Hz for standard ECG recordings (Kligfield et al., 2007). It is important to bear these differences in mind when comparing measurements obtained from standard resting ECG and Holter recordings.

The majority of studies applied the Bazett formula to correct QT measurement, whereas only two studies used multiple correction formulae (Hermans et al., 2018; Vaglio et al., 2008). Interestingly, Couderc et al. (2011) showed specificity of the Bazett formula in the LQT2 model, and Fridericia formula in their moxifloxacin aLQTS model. The Bazett formula is most commonly used in clinical practice, owing to its simplicity and association with outcome data (Waddell‐Smith et al., 2017). However, numerous formulae exist which appear to perform better at different specific heart rate ranges (Indraratna et al., 2019) and for different LQTS genotypes (Barsheshet et al., 2011). Given no formula is universally applicable and all have limitations, variability in the QT correction process further limits the diagnostic capability of this ECG biomarker alone and reinforces the value of integrating evaluation of TWM (Indraratna et al., 2019; Waddell‐Smith et al., 2017).

### 4.1 Approaches to measuring T wave morphology

The included biomarkers identified across all studies can be categorized as TW‐specific fiducial points (Tpeak‐Tend and J‐Tpeak intervals, amplitude, duration), architectural patterns on an independent (flat, asymmetric, notched) or combined (MCS, PCA) basis, functions, and integrals derived from TW slopes (sigmoidal and polynomial functions, RI), vectorcardiographic biomarkers; and unique measures pertaining to the Mayo Clinic's *proprietary T wave analysis program* (Sugrue et al., 2015). An important limitation in evaluating this literature is that abnormal TWM acted as exclusion criteria across several studies (Hermans et al., 2018; Immanuel et al., 2016; Sugrue et al., 2016; Sugrue, Noseworthy, et al., 2017; Sugrue, Rohatgl, et al., 2017), raising the possibility of missing relevant TWM changes despite the intention of standardizing the data being analyzed.

#### 4.1.1 T wave‐specific fiducial points

Tpeak‐Tend is a measure of spatial dispersion of ventricular depolarization, representing late repolarization, understood to predict arrhythmic risk (Antzelevitch et al., 2019; Johannessen et al., 2014). The mechanism is explained by transmural or global myocardial dispersion of repolarization refractoriness, introducing a vulnerable window for early afterdepolarization‐induced extrasystoles to be captured, precipitating TdP (Antzelevitch et al., 2019). The arrhythmogenic potential of this biomarker has been demonstrated in several pathophysiological conditions, including LQTS, reinforcing its unquestionable role in identifying individuals at high risk of arrhythmic SCD (Antzelevitch et al., 2019; Shimizu et al., 2002; Takenaka et al., 2003; Topilski et al., 2007).

Irrespective of its overwhelming value as an independent risk factor for arrhythmogenesis, four studies demonstrated important clinical applications of Tpeak‐Tend in combination with other TWM biomarkers (Sugrue, Noseworthy, et al., 2017; Sugrue et al., 2016; Johannessen et al., 2014: Vaglio et al., 2008). Sugrue, Noseworthy, et al. (2017) demonstrated lead‐specific discrimination of aLQTS from cLQTS in V_5_ based on prolonged Tpeak‐Tend, and shallower TW right slope and smaller COG. Similarly, lead‐specific discrimination of cLQTS and concealed cLQTS from control in V_6_ was achieved using the same analysis model based on a longer Tpeak‐Tend, in addition to left slope of the TW and COG *x* axis (Sugrue et al., 2016). Vaglio et al. (2008) used Tpeak‐Tend in combination with two other scalar ECG parameters, TW magnitude, and QTpeak, to differentiate cLQTS individuals from controls.

Johannessen et al. (2014) evaluated early and late repolarization using J‐Tpeak and Tpeak‐Tend, respectively, in a drug‐induced multichannel block model involving dofetilide, quinidine, ranolazine, and verapamil. Pure hERG block with dofetilide prolonged both the J‐Tpeak and Tpeak‐Tend, compared to additional calcium and late sodium blockade which preferentially reduced the J‐Tpeak. QTc prolongation was reported as occurring equally in dofetilide‐induced hERG blockade and multichannel block with quinidine, the J‐Tpeak, and Tpeak‐Tend played a key role in differentiating pure hERG from multichannel block in this aLQTS cohort. By comparison, Tpeak‐Tend did not demonstrate independent prognostic value for arrhythmogenesis risk stratification in a cLQTS cohort of LQT2 genotype positive individuals with normal QTc intervals, compared to healthy family controls (Platonov et al., 2018). Similarly, Immanuel et al. (2016) showed there was no difference in Tpeak‐Tend despite the QTc interval being found to be marginally longer in cLQTS individuals compared to controls.

Heart rate correction is emerging as an important consideration in assessing some TWM fiducial point parameters, particularly the J‐Tpeak (Hnatkova, Vicente, Johannesen, Garnett, Straus, et al., 2019). J‐Tpeak effects are of particular interest because studies showing that if QT‐prolonging drugs affect both the J‐Tpeak and Tpeak‐Tend, rather than the Tpeak‐Tend alone, they are more likely to purely block the delayed potassium rectifier current (IKr) resulting in proarrhythmic effects (Hnatkova, Vicente, Johannesen, Garnett, Straus, et al., 2019). Drugs solely prolonging the Tpeak‐Tend have been shown to impact multiple ion channels, thus alleviating the arrhythmogenic effects of IKr blockade (Hnatkova, Vicente, Johannesen, Garnett, Straus, et al., 2019). Thus, to accurately assess the J‐Tpeak so as to accurately evaluate proarrhythmic risk of QT‐prolonging drugs, correction of the fiducial point for heart rate is needed (Hnatkova, Vicente, Johannesen, Garnett, Stockbridge, & Malik, 2019). Hnatkova, Vicente, Johannesen, Garnett, Straus, et al. (2019) developed correction formulae for both the J‐Tpeak and the JT50 interval, referring to the intervals between the J point and the median point of the area under the TW. These formulae were found to be efficacious at increasing the accuracy of interpreting the two selected fiducial points and thus delineate IKr from multichannel blockade, with the specific application for clinical pharmacology studies resulting in drug‐induced heart rate changes up to 10 beats per minute (Hnatkova, Vicente, Johannesen, Garnett, Straus, et al., 2019).

TW amplitude and duration were selected fiducial points in work by Moss et al. (1995). T‐duration was found to be longest, and T‐amplitude was most reduced in cLQTS individuals who were chromosome 11 and chromosome 7 mutation positive, respectively (Moss et al., 1995). While these biomarkers were able to identify cLQTS positive individuals, contemporary studies have applied more comprehensive models which have been developed since this initial innovative study was performed.

#### 4.1.2 T wave architectural patterns

Architectural patterns of flatness, asymmetry, and notching were incorporated across five studies (Graff et al., 2009; Graff et al., 2010; Platonov et al., 2018; Porta‐Sanchez et al., 2017; Vicente et al., 2015). Vicente et al. (2015) reported on these TW metrics in their drug‐induced multichannel block model, which also utilized dofetilide, quinidine, ranolazine, and verapamil. Relative channel block and ECG biomarkers were presented for each drug, with a linear‐mixed effects model performed to assess flatness and asymmetry (dimensionless units), and logistic regression model used for quantifying notching (%), in relation to maximum drug concentration (Vicente et al., 2015). Notching was the predominant TW metric change associated with substantial hERG blockade for both dofetilide, 55% notching (55% hERG block), and quinidine, 69.7% notching (71% hERG block; Vicente et al., 2015).

Platonov et al. (2018) used architectural TW biomarkers to identify the risk of cardiac events in LQT2 genotype positive individuals with normal QTc intervals. TWs in leads II and V_5_ were classified as either normal, or abnormal based on being broad, flat, notched, negative, or biphasic (Platonov et al., 2018). A composite rating of TW abnormality was relied upon for risk stratification, rather than assessing the impact of specific architectural patterns, with the multivariate analysis indicating an association between abnormal TWM and both female sex, and pore location LQT2 mutations in men (Platonov et al., 2018).

An alternative means of utilizing TW architectural patterns for diagnostic purposes is the MCS (Graff et al., 2009; Graff et al., 2010; Porta‐Sanchez et al., 2017). Porta‐Sanchez et al. (2017) determined MCS, based on PCA and PCA‐2 values, using the formula: *MCS = 1.6* × *flatness + asymmetry + notch*. The architectural components were calculated as follows: flatness was based on 1‐kurtosis of TW area, asymmetry relied upon evaluation of slope profile and duration of ascending and descending components of the TW, and notching was captured from the inverse signed radius of curvature (Porta‐Sanchez et al., 2017). This model was precise enough to diagnose cLQTS compared to control, distinguish between LQT1 and LQT2 genotypes, and detect genotype positive cLQTS cases in the context of a normal QTc (Porta‐Sanchez et al., 2017).

Graff et al. (2010) and Graff et al. (2009) applied a similar MCS formula to their work in both cLQTS and aLQTS cohorts: *MCS = asymmetry + notch + 1.6* × *flatness*. Determination of flatness similarly used the kurtosis measure, while asymmetry used first derivatives (i.e., average of the square of the difference between the slopes) of the ascending and descending parts of the TW (Graff et al., 2009; Graff et al., 2010). Notching was also assessed using a curvature signal, which was calculated from the first and second derivatives of TWs, prior to magnitude being measured on the basis of TW amplitude (Graff et al., 2009; Graff et al., 2010).

Both studies (Graff et al., 2009; Graff et al., 2010) which applied this model demonstrated efficacy of MCS over QTc in an aLQTS cohort of healthy individuals prescribed sotalol, for doses of 160 mg and 320 mg. Graff et al. (2009) then compared TW changes in their healthy cohort post administration of sotalol with known LQT2 positive individuals, demonstrating similarities in MCS despite differences in QTc. The MCS approach is arguably more comprehensive than evaluating for the presence of a single architectural component, as it ascribes equal importance to each architectural variable, thus including the different aspects of abnormal repolarization that they bring to the composite measure (Graff et al., 2009; Graff et al., 2010). Another benefit is that it has been shown to have efficacy in both cLQTS and aLQTS groups.

#### 4.1.3 T wave slope functions and integrals

Work by Immanuel et al. (2016) expanded on the evaluation of traditional ECG parameters of the QT interval, TW amplitude; and Tpeak‐Tend, by interrogating the upslope and downslope of the TW with either Boltzmann sigmoid functions or polynomial fitting. Performance of these curve fitting techniques was comparable, demonstrating greater efficacy than conventional measures in distinguishing LQTS from control, and differentiating between LQT1 and LQT2 individuals (Immanuel et al., 2016). Beyond developing this unique and efficacious modality for assessing the TW, the model was a fully automated computerized classification approach using a NNC technique, allowing for analysis of Holter data which provided a large set of beats in a variable range of heart rates. This overcame one limitation of preceding work which largely relied on manual measurement or semiautomated analysis of digitized ECGs, which were dependent on resting recordings which did not account for dynamic changes (Immanuel et al., 2016).

Automated polynomial curve fitting has since been applied by Hermans et al. (2020) using Hermite‐Gauss polynomials, whereby TW characteristics were identified and extrapolated in an unbiased manner then added to the comprehensive SVM “extended” model. The model's capacity for correctly classifying cLQTS in the context of a normal QTc was 84%, which correlated well with assessment by a “QT expert” cardiologist showing 87% agreement (Hermans et al., 2020). The specificity for TW biomarkers based on polynomial modeling in individuals with a normal QTc was comparable with findings of Immanuel et al. (2016), which showed a detection rate of 90%.

In addition to TWM characterization being fully automated, the SVM models enabled a machine learning facet allowing for 10‐fold cross‐validation involving partitioning of data into 10 subsets to facilitate training of the first 9 subsets and testing on the tenth (Hermans et al., 2020). Robustness was then confirmed on a second set of data, prior to confirmation of performance using measures of sensitivity, specificity, and accuracy (Hermans et al., 2020). Sigmoid modeling, achieved using the RI, was able to differentiate between hERG versus KvLQT1 mutations (Kanters et al., 2004) and LQT2 versus LQT1 cohorts (Vaglio et al., 2008), however was applied in two studies only and has been replaced by contemporaneous modeling.

#### 4.1.4 Vectorcardiographic biomarkers

Vectorcardiographic biomarkers varied across the five studies (Couderc et al., 2011; Hermans et al., 2018; Johannessen et al., 2014; Vaglio et al., 2008; Vicente et al., 2015) in which they were used, however their efficacy in characterizing repolarization was clearly demonstrated. Discrimination of cLQTS from controls reached 90% in one study (Vaglio et al., 2008), in addition to enhanced diagnostic accuracy, with an improvement in sensitivity from 69.4% to 82% and specificity from 82.9% to 86.1% (Hermans et al., 2018). Vector quantities determined the effect of selected drugs on normal repolarisation in healthy individuals assessed in concentration‐dependent analyses in aLQTS models, differentiating the degree and type of channel blockade (Couderc et al., 2011; Johannessen et al., 2014; Vicente et al., 2015). Couderc et al. (2011) showed T‐roundness was equally as effective as QTc at determining risk of cardiac events, in addition to doing so independently (*p* < .007).

#### 4.1.5 Proprietary T wave analysis program

While each method of analyzing TWM has demonstrated some merit in the diagnosis of LQTS, one significant challenge pertains to standardization of all relevant TW interrogation methodologies into a single model which can be applied to both cLQTS and aLQTS cohorts. Sugrue and colleagues' (Sugrue et al., 2015; Sugrue et al., 2016; Sugrue, Noseworthy, et al., 2017; Sugrue, Rohatgl, et al., 2017) novel, *proprietary T wave analysis program*, produced using a MATLAB package, enabled automatic ECG feature extraction which are detected by a Bayesian statistical peak delineation algorithm. TW components included the specific fiducial points of amplitude and Tpeak‐Tend, left and right slopes, enclosed TW area; and the unique measure of COG allowing for determination of the *x*/*y* coordinates of the COG of the first and last 25% of the TW (Sugrue, Rohatgl, et al., 2017).

The robustness of this model has been tested in an aLQTS cohort following initiation of dofetilide and sotalol (Sugrue et al., 2015), cLQTS cohort distinguishing concealed from manifest LQTS (Sugrue et al., 2016); and differentiating aLQTS from cLQTS (Sugrue, Noseworthy, et al., 2017). Furthermore, the same model has also been shown to predict the risk of breakthrough arrhythmic events through Kaplan–Meier methods and use of C‐statistics, including genotype‐specific subgroup analyses (Sugrue, Rohatgl, et al., 2017). Despite the diverse clinical utility of this novel model, it does not incorporate key proven methods of TW interrogation, including sigmoidal and polynomial functions, MCS; and vectorcardiographic biomarkers.

With the establishment of the *International Long‐QT Syndrome registry* (Moss & Schwartz, 2005; Vandenberg et al., 2017) and *CredibleMeds* initiative (Credible Meds, 2020), the impetus for instituting collaboration in the LQTS field on a global scale is clear. A case has been made to further enhance the utility of the *proprietary T wave analysis program* by incorporating the additional analytical process of automated polynomial functions, introducing complex matters surrounding intellectual property but also the need to test the subsequent new model across the same clinical circumstances already investigated (Hermans et al., 2020). In considering the catastrophic cost associated with LQTS, that being SCD, a collegiate approach is an essential step forward in optimizing the process of risk stratification through standardization of TWM assessment. This includes the process of dissemination, which could easily be achieved through publication in international guidelines. Practical uptake of TWM measurements requires a substantial paradigm shift.

### 4.2 Application of T wave morphology to arrhythmia risk prediction

QTc prolongation continues to be considered the most useful warning signal for TdP, with the risk of life‐threatening arrhythmias increasing by 15% for each 10 ms increment in QTc duration (Giudicessi et al., 2019; Mazzanti et al., 2018; Schwartz & Woosley, 2016). It has even been proposed that the QTc could play an evolving role for risk stratification in the future, acting as a vital sign monitored over the lifespan with the aid of mobile ECG devices screening for the impact of QT‐aggravating factors (Giudicessi et al., 2019). Critical to this transition in the diagnostic approach of LQTS is overcoming the challenge of identifying repolarization abnormalities in the presence of a normal QTc. Herein lies the importance of incorporating the sophisticated methods of TWM analysis presented in this review. Practical challenges surrounding their application clinically must be addressed.

The process of ECG recording and digitization allowing for uploading a centralized database, to which standardized TWM analysis programming can be applied, is critical. Integrating such a system with the electronic medical record and medication chart would enable capturing associated QT‐prolonging factors, thus determining mortality risk as has been described using the *QT alert system* (Haugaa et al., 2013; Schwartz & Woosley, 2016; Sugrue, Noseworthy, et al., 2017). Given the computational nature of this proposed clinical process, and proven application of neural network (Immanuel et al., 2016) and machine learning (Hermans et al., 2018; Hermans et al., 2020) analyses to TW models in LQTS, the integration of artificial intelligence (AI) programming should be considered to enhance diagnostic capabilities through deep learning over time (de Marvao et al., 2020; Johnson et al., 2018).

### 4.3 Wearable devices and artificial intelligence

Proof of concept work has suggested a role for AI in diLQTS (Attia et al., 2018). AI was applied to surface ECG biomarkers to predict dofetilide plasma concentrations, which was shown to be more efficacious than QTc (Attia et al., 2018). Preliminary data has shown similar promise in cLQTS cohorts using a convolutional neural network to distinguish genotype positive individuals, independent of QTc (Bos et al., 2018; Hajimolahoseini et al., 2019). As the clinical utility of this technology continues to improve, assimilation into TW analysis programs is likely to enhance the sophistication of such models. The proposed benefit being maximizing diagnostic accuracy through earlier detection and the institution of risk mitigating interventions.

With the advent of mobile devices being incorporated into clinical care within the cardiac electrophysiology realm, the capacity for longer periods of monitoring in an inexpensive and less invasive manner is made possible (de Marvao et al., 2020). This builds on limited data acquisition enabled with static 12 lead ECGs, telemetry during hospital admissions; and Holter monitors which are often only worn for 24 hours.

Castelletti et al. (2018) showed that a wearable monitor producing a single lead ECG can reliably assess the QTc, in both healthy controls and a cLQTS cohort, compared to 12 lead ECG Holter monitor traces. This technology facilitates circumventing the clinical problem of long‐term monitoring in the first few weeks (i.e., up to a total of 30 days) of therapy with torsadogenic drugs for arrhythmogenic QT lengthening, allowing for prompt determination of safe prescription or the need to interrupt therapy early (Verrier, 2018). This is relevant to whether there is an identified underlying genotype‐specific cLQTS diagnosis or not, which is known to increase the risk of diLQTS in 30% of affected individuals causing cumulative QT prolongation (Castelletti et al., 2018; Itoh et al., 2016; Schwartz & Woosley, 2016; Strauss et al., 2017). Further, this approach may also be used in tracking beneficial QTc changes in response to different treatment modalities used in cLQTS patients (Verrier, 2018).

Despite mobile devices only recording from a single lead, preliminary results on information extracted are promising (Bos et al., 2018; Castelletti et al., 2018). However, it has recently been recommended that at least three leads be assessed when using mobile handheld devices to review the QTc, due to the risk of QTc underestimation if a single position is used (Cheung et al., 2020).

Regardless of the means of ECG data acquisition, key stages of development will be enabling digitization and application of TW analysis programming which can then be added to the patient's electronic medical record, allowing for remote access by the relevant treating physician and contemporaneous comparison, which will enhance the utility of this common and essential clinical investigation. As such, specific education‐based curriculums of relevant clinicians will undoubtedly be required, using the example of transitioning the simple clinical skill of ECG lead placement and recording toward the aforementioned digitized processes.

This is indeed an exciting time for the delivery of healthcare, particularly in relation to the possibilities allowing for mitigating the risk of SCD in the setting of LQTS. Another interesting perspective relates to economics, specifically performing relevant cost benefit analyses as this technology is increasingly applied in a clinical capacity. In considering the ultimate cost associated with this devastating disease process, that being the loss of life secondary to this devastating disease process, at this stage striving to transition relevant translational research into a clinically meaningful tool which can be applied to patients in the real world is essential

## 5 CONCLUSION

TWM biomarkers provide a substantial amount of supplementary information, aiding the diagnosis of abnormal repolarization and risk stratification process in both cLQTS and aLQTS. Current major challenges relate to a lack of standardization in programming and biomarkers used which are applicable to both cLQTS and aLQTS groups, ECG digitization and automated analyses, integration of AI; and translation into clinical settings. Despite these obstacles, there is great promise in this ever‐evolving area in relation to QTc monitoring. This progress provides the foundations for integrating TWM biomarkers into the process of risk stratification, to achieve the fundamental goal of preventing the devastation associated with SCD.

## CONFLICT OF INTEREST

The authors declare that the research was conducted in the absence of any commercial or financial relationships that could be construed as a potential conflict of interest.

## AUTHOR CONTRIBUTIONS

DT performed the literature review and collation of data for review. DT, MP, RS, JV and AH contributed to planning, writing and editing of the manuscript and figures.

## ETHICS STATEMENT

Institutional ethics approval was not required because the data analyzed are from previous published studies, in which informed consent, or a waiver of consent was obtained. The PRISMA guidelines have been utilized for reporting purposes.

## Data Availability

The data that supports the findings of this study are available in the supplementary material of this article
